# The Endothelial Glycocalyx and Organ Preservation—From Physiology to Possible Clinical Implications for Solid Organ Transplantation

**DOI:** 10.3390/ijms22084019

**Published:** 2021-04-13

**Authors:** Simon Mathis, Gabriel Putzer, Stefan Schneeberger, Judith Martini

**Affiliations:** 1 Department of Anaesthesiology and Critical Care Medicine, Medical University of Innsbruck, 6020 Innsbruck, Austria; simon.mathis@i-med.ac.at (S.M.); gabriel.putzer@i-med.ac.at (G.P.); 2 Department of Visceral, Transplant and Thoracic Surgery, Medical University of Innsbruck, 6020 Innsbruck, Austria; stefan.schneeberger@i-med.ac.at

**Keywords:** glycocalyx, syndecan-1, heparan sulfate, solid organ transplantation, static cold storage, normothermic machine perfusion

## Abstract

The endothelial glycocalyx is a thin layer consisting of proteoglycans, glycoproteins and glycosaminoglycans that lines the luminal side of vascular endothelial cells. It acts as a barrier and contributes to the maintenance of vascular homeostasis and microperfusion. During solid organ transplantation, the endothelial glycocalyx of the graft is damaged as part of Ischemia Reperfusion Injury (IRI), which is associated with impaired organ function. Although several substances are known to mitigate glycocalyx damage, it has not been possible to use these substances during graft storage on ice. Normothermic machine perfusion (NMP) emerges as an alternative technology for organ preservation and allows for organ evaluation, but also offers the possibility to treat and thus improve organ quality during storage. This review highlights the current knowledge on glycocalyx injury during organ transplantation, presents ways to protect the endothelial glycocalyx and discusses potential glycocalyx protection strategies during normothermic machine perfusion.

## 1. Introduction

Organ transplantation is the gold standard for the treatment of refractory heart failure, end-stage lung disease, acute and chronic liver failure, and end-stage renal disease [1,2,3,4]. Due to organ scarcity, an increasing number of extended-criteria organs are being considered for transplantation [5,6]. These organs are particularly vulnerable and suffer additional damage during retrieval, storage and reperfusion. Although cold storage on ice limits organ damage during transportation and storage, grafts become acidotic. The subsequent reperfusion induces the formation of reactive oxygen species and excessive influx of calcium into the cells, leading to cell swelling, destruction of cell integrity and cell death [7]. Ischemia Reperfusion Injury (IRI), an acute sterile inflammation of the graft after reperfusion, negatively impacts on organ quality and is associated with poorer outcomes in solid organ transplantation [8,9].

The endothelial glycocalyx is an important target in the complex pathophysiological process of IRI. This thin layer, discovered more than 50 years ago [10] lines the inner wall of the endothelium consisting of proteoglycans and glycosaminoglycans. It has been identified as acting as a barrier structure which modulates the adhesion of leukocytes and platelets to the endothelium, participates in coagulation and functions as a mechanotransducer by transmitting shear stress to subendothelial structures [11,12,13,14]. Its destruction appears to play a central pathophysiological role in the development of IRI in shock, myocardial infarction, stroke, traumatic blood loss and during solid organ transplantation [15,16,17,18,19,20,21]. Various pharmacological and non-pharmacological therapeutics have been identified that may prevent glycocalyx destruction or restore glycocalyx integrity [22,23]. Since cold organ storage during transportation minimizes the biological activity of organs, such interventions are not feasible since pharmacological treatment during storage on ice is largely ineffective. In recent years, alternative technologies which create a more physiological environment for grafts have emerged to clinical realization [24]. Normothermic organ perfusion may not only evolve as an ideal organ preservation method in transplantation, but may also offer the possibility to assess and eventually modify glycocalyx integrity and functionality.

This narrative review summarizes the current knowledge on glycocalyx changes during solid organ transplantation and discusses present and potential future options for glycocalyx protection and repair mechanisms.

## 2. Structure of the Glycocalyx

**Figure 1:**
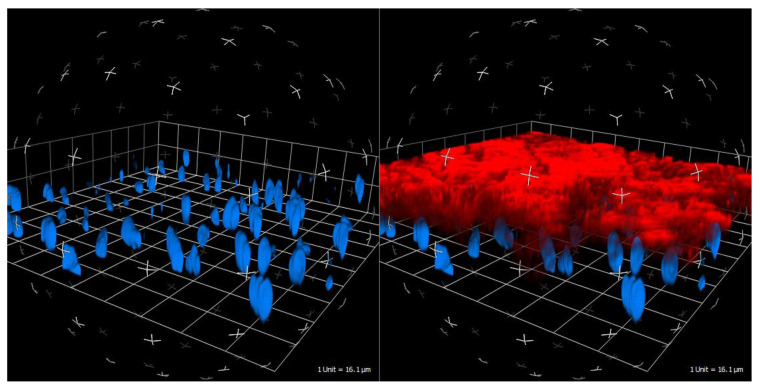
Real time live confocal visualization of the glycocalyx (in red via wheat germ agglutinin) and endothelial cell nuclei (in blue via HOECHST stain). Porcine blood vessel, 40× water immersion objective.

The endothelial glycocalyx is a complex 0.1–1 µm thick layer that lines the luminal surface of endothelial cells [22]. As early as 1940, Danielli postulated that a protein layer coats the endothelium [25]. However, direct imaging evidence in electron microscopy was not achieved until a few years later by staining with ruthenium red [10]. Proteoglycans (e.g., syndecans) and glycoproteins form the backbone of this structure and are anchored in the endothelial cells [26,27]. Heparin and chondroitin sulfate glycosaminoglycans covalently bind to these transmembrane proteins, thereby forming a negatively charged matrix where plasma components such as albumin and orosomucoids accumulate [28,29] (Figure 1). Due to vivid crosstalk between different constituents of the glycocalyx, a physiologically active stratum is formed, also referred to as the endothelial surface layer [26,30].

If the glycocalyx is damaged by different mechanisms, glycocalyx-shedding products can be measured in the plasma. Especially, syndecan-1 and heparan sulfate are components of the endothelial glycocalyx that have increased plasma concentrations after glycocalyx injury. Different studies have shown that the amount of glycocalyx-shedding products correlates with the severity of the underlying pathological condition [31,32]. Jung et al. showed in cardiogenic shock patients that increased levels of syndecan-1 at admission significantly correlated with poor outcome, thus suggesting that glycocalyx integrity and preservation may be important factors in the maintenance of physiological cardiovascular function [32].

## 3. Function of the Endothelial Glycocalyx and Pathophysiological Implications

Even prior to the identification of the biochemical structure of the glycocalyx, the presence of a peripheral layer of plasma surrounding the red blood cell column was observed, and indicated the anatomical location of the glycocalyx [33,34]. The finding by Pries et al., namely that the flow in microvessels is significantly lower than the flow in glass capillaries with the same diameter, demonstrated that the presence of this plasma layer has a significant influence on flow properties of blood in the vascular system [35]. It had previously been noticed, that the haematocrit in capillaries (i.e., the fractional volume of red blood cells within a capillary) was significantly lower than the core haematocrit and the arteriolar haematocrit, which was attributed to the presence of this slow-moving cell-free plasma layer adjacent to the endothelial cell lining [34]. This layer, later identified as the glycocalyx protruding into the vessel lumen, is the reason why red blood cells flow in the center of the vessel [36]. Studies have estimated that the presence of this plasma layer reduces the mean optical haematocrit in microvessels to 25% of core haematocrit. Further studies have shown that the thickness of this layer can be reduced by treatment with heparinase, which allows the red blood cell column to flow closer to the vascular wall [37]. This increases the width of the red cell column, thereby increasing the fractional volume of red blood cells in the capillary.

It has become increasingly clear that the endothelial glycocalyx mainly regulates vessel wall permeability for fluids and proteins. Contrary to the Starling principle, postulated in the 19th century, fluid exchange via the endothelium is not only driven by the hydrostatic and oncotic pressure prevailing in the vessel and in the interstitium, respectively. Instead, the equilibrium appears to be established by means of the endothelial glycocalyx rather than by the entire capillary wall, thereby reducing fluid filtration into the interstitium [38]. Studies have shown that enzymatic destruction of the endothelial glycocalyx increases hydraulic conductivity leading to the increased clearance of albumin in rat glomeruli and to the formation of myocardial edema [39,40,41].

Moreover, the endothelial glycocalyx prevents endothelial cells from interacting with cellular blood components. Several experiments have demonstrated that the destruction of the glycocalyx leads to the increased adhesion of red blood cells, leukocytes and platelets to the endothelium, thus leading to perfusion deficits [42,43]. Interestingly, a thick (“healthy”) glycocalyx reflects efficient perfusion of the microvascular bed, whereas a thin (“risk”) glycocalyx is associated with poor perfusion [44].

The endothelial glycocalyx also acts as a mechanosensor of the vessel wall, transferring mechanical forces, e.g., shear stress, to the endothelial cell. It is well known that endothelial cells produce nitric oxide (NO) in response to shear stress, which induces vasodilatation due to the relaxation of vascular smooth muscle cells and leads to improved perfusion of downstream microvascular beds [45]. Although the exact mechanism of mechanotransduction is not clear, there is evidence that the endothelial glycocalyx is involved [46,47]. This is also supported by several studies which show that NO release is reduced after the glycocalyx is destroyed by hyaluronidase [48].

The endothelial glycocalyx is an enzymatically highly active region. The different endothelial glycosaminoglycans serve as docking points for many plasma-derived molecules such as growth factors, antithrombin, cell adhesion molecules and different cytokines [49]. By binding enzymes and their ligands, the glycocalyx is the anatomical region where numerous enzymatic processes take place [49,50]. One of the most important ones is the binding of antithrombin. Antithrombin binds to heparan sulfate anchors in the glycocalyx, leading to a heparin-like effect that prevents clot formation on the endothelial surface and mediates a profibrinolytic and antithrombotic milieu [51]. It has been shown that the degree of destruction of the glycocalyx, due to pathological processes such as sepsis, is a predictive marker for the development of disseminated intravascular coagulation; which is characterized by thrombosis, on the one hand, and coagulopathy with increased bleeding, on the other [52,53]. Shedding of the glycocalyx may result in systemic heparin-like effects, resulting in a status of auto-heparinisation, which may play a major role in the development of trauma-induced coagulopathy and is also discussed as a potential mechanism for reperfusion-coagulopathy during orthotopic liver transplantation in cirrhotic patients [13,54,55].

## 4. The Effect of Solid Organ Transplantation on the Endothelial Glycocalyx

IRI is a key driving factor for graft damage in heart, lung, liver and kidney transplantation [56,57,58,59]. Recent studies have suggested that damage to the endothelial glycocalyx significantly contributes to the development of IRI [21,60,61]. Schiefer et al. found significantly higher plasma levels of syndecan-1 in liver graft recipients after transplantation than before transplantation, indicating destruction of the endothelial glycocalyx [21]. The same study group showed that the levels of syndecan-1 measured in the preservation fluid of liver grafts significantly correlated with hepatic injury markers in the preservation fluid, such as alkaline phosphatase, aspartate aminotransferase and lactate. These findings suggest that glycocalyx degradation occurs in proportion to liver graft injury [62]. Furthermore, it was determined that effluent syndecan-1 concentrations were greater in recipients who had developed an early allograft dysfunction (EAD) than in patients who had not [62]. Taken together, these data suggest a possible role for glycocalyx degradation markers as monitoring parameters for liver graft function early after transplantation.

Another parameter indicating glycocalyx destruction is heparan sulfate. However, Passov and co-workers recently showed that heparan sulfate behaves differently from syndecan-1 during liver transplantation [60]. Whereas syndecan-1 plasma levels strongly increase immediately after reperfusion of the liver graft, plasma levels of heparan sulfate significantly decrease compared to pre-transplant levels. The reason for this finding is still unclear, but the authors assume that heparan sulfate is absorbed into the liver, presumably to restore the destroyed glycocalyx [60].

Destruction of the glycocalyx has also been demonstrated in kidney grafts. After kidney transplantation from donation after circulatory death (DCD) donors, the so-called red blood cell exclusion zone of peritubular capillaries, measured by sidestream darkfield microscopy, is significantly thinner when compared to kidneys from living donors, which indicates destruction of the endothelial glycocalyx during ex situ kidney preservation. In addition, the arteriovenous gradients of syndecan-1 and heparan sulfate were greater in DCD kidneys than in kidneys from living donors [63].

A significant increase in syndecan-1 and heparan sulfate was also observed in lung transplant recipients. The clinical relevance of glycocalyx damage is indicated by the finding that primary graft dysfunction, defined as PaO2/FiO2 < 300, was associated with higher plasma syndecan-1 levels 72 h after transplantation [61]. Syndecan-1 plasma levels during the first four days after transplantation also showed a correlation with length of ICU stay and duration of ventilation.

Glycocalyx damage in donors predicts poor function after lung transplantation. Sladden et al. found that the only donor factors increasing graft acceptance for lung transplantation were high PaO2 and low plasma hyaluronan levels; another known glycocalyx destruction parameter. Each 10 ng/mL decrease in hyaluronan levels in donor plasma was equivalent to a 4% increase in the likelihood of the graft being accepted for transplantation. In addition, primary graft dysfunction, characterized by PaO2/FiO2 < 300 during the first 24 h after transplantation, was associated with significantly higher syndecan-1 levels in the respective lung donors [61].

## 5. Glycocalyx-Protective and Restoration Strategies

Based on the large body of literature characterizing the mechanisms of IRI on the endothelial glycocalyx, it seems promising to investigate interventions, aiming to protect or even restore the glycocalyx. Various drugs that may protect and/or restore the endothelial glycocalyx have been tested in animal studies, while human trials are still lacking. Glycocalyx-protective strategies have been investigated during major surgery [64] and the results indicated that preventive measures towards glycocalyx destruction may be effective regardless of the mechanism of damage. The following paragraphs will summarize current knowledge about clinical approaches, aiming to protect the glycocalyx during surgery. It is addressed with a focus on the main findings of experimental animal studies to preserve and restore endothelial glycocalyx during IRI of the graft.

In intensive care and perioperative medicine, adequate fluid therapy is a prerequisite for maintaining organ perfusion and oxygenation and for preventing tissue hypoxia. Due to its barrier function, the intact endothelial glycocalyx plays an important role in physiological fluid homeostasis and impedes rapid large transmural fluid shifts from the intravascular to the extravascular compartment. However, the endothelial glycocalyx is particularly sensitive to fluid loading conditions, i.e., hypervolemia [65]. Berg et al. showed that in healthy volunteers the “on top” administration of 1000 mL of Ringer’s acetate led to an increase in plasma hyaluronan levels, thus indicating damage to the glycocalyx [66]. Chappell et al. compared the “on top” administration of 1250 mL of 6% hydroxyethyl starch prior to surgery, with patients who underwent normovolemic hemodilution, i.e., by withdrawing 1250 mL of blood and administering 1250 mL of 6% hydroxyethyl starch simultaneously [67]. Hypervolemia, as induced in the first patient group, was associated with a release of atrial natriuretic peptide and an increase in glycocalyx-shedding parameters in the plasma. Atrial natriuretic peptide, in addition to its diuretic and vasodilatory properties, mediates a fluid shift from the intravascular to the extravascular space and induces shedding of the glycocalyx [68,69]. Similar results were obtained in a study comparing a liberal fluid regime (15 mL/kg/h) during laparoscopic cholecystectomy and a restrictive fluid regime (1 mL/kg/h). Glycocalyx shedding was significantly increased after liberal fluid administration as compared to the restrictive protocol [65]. In summary, these studies suggest that fluid replacement protocols which are aimed at the maintenance of normovolemia and avoidance of hypervolemia seem to support physiological glycocalyx function.

The question as to which intravenous solution should be used for fluid replacement and volume therapy dates back to the early 1940s and is still controversially discussed [70,71,72,73,74,75,76]. In view of their effect on the endothelial glycocalyx, colloids seem to better protect its physiological functioning than crystalloids. Torres et al. showed, in a hemorrhagic shock model in rats, that resuscitation with crystalloids seems to be associated with greater destruction of the glycocalyx than resuscitation with fresh frozen plasma or albumin [77]. Additionally, microvascular barrier function, as assessed by measurements of microvascular permeability, seems to be better preserved with infusions of colloids than with crystalloid solutions [78]. Hence, the type and amount of volume replacement fluids seem to play an important role in maintaining physiological glycocalyx function.

Although none of these studies explicitly refer to solid organ transplantations, it is reasonable to conclude that most of these physiological conditions should be pursued during transplantation.

Hyperglycemia is a common phenomenon in the perioperative phase of transplantation medicine. Besides organ-related specific factors (e.g., destruction of hepatocytes following IRI leading to a systemic influx of glucose during liver transplantation [79], and the high prevalence of impaired glucose tolerance in kidney transplant patients [80]), the administration of glucocorticoids, vasoactive drugs and the surgical–stress reaction lead to metabolic imbalances and hyperglycemia [81,82,83]. Perioperatively, a blood–glucose level of 5–10 mmol/L is recommended, which can be achieved with the administration of insulin [84]. It has been shown that persistent hyperglycemia (glucose concentrations of 16 mmol/L over six hours) induces a reduction in total glycocalyx volume by 50% in healthy volunteers, while plasma concentrations of hyaluronan significantly increased [85]. Similar results were found in animal and in vitro studies [86,87,88].

Additionally, the choice of the anesthetic technique seems to have an influence on the integrity of the endothelial glycocalyx. It has already been shown that neuroaxial anesthetic techniques, such as spinal or epidural anesthesia, better preserve glycocalyx structure and function than general anesthesia [89]. However, these techniques are not feasible during transplant surgery. When assessing general anesthesia, however, it was shown that the inhaled anesthetic sevoflurane appears to generate glycocalyx-protective properties in various organs. In an IRI model on rat livers, animals were anesthetized with either intravenous ketamine or inhaled sevoflurane before their livers were removed and subsequently reperfused. During reperfusion, glycocalyx-shedding parameters were significantly lower in the perfusate of the sevoflurane group than in the ketamine group, indicating reduced IRI and reduced glycocalyx destruction as confirmed by electron microscopy imaging [90]. Similar results were obtained in a study of lung autotransplantation, where pigs were anaesthetized with either propofol or sevoflurane [91]. After reperfusion, rates of glycocalyx degradation products were higher in the plasma of propofol animals than in animals in the sevoflurane group [91]. A similar effect of sevoflurane on the glycocalyx has also been shown in isolated guinea pig hearts, where the protective effects of sevoflurane were evident regardless of whether sevoflurane was added to the gas mixture before ischemia or after reperfusion [92,93]. In human liver transplantation, however, no positive effects of sevoflurane or other inhalative anesthetics on the glycocalyx have yet been demonstrated [21]. Additionally, other clinical studies comparing the effects of sevoflurane and propofol have shown no benefit so far [94]. Taken together, sevoflurane and propofol may have beneficial effects on the glycocalyx, but the magnitude of these effects does not appear to be significant in major surgery.

## 6. Alternative Organ Preservation Strategies and Their Influence on the Glycocalyx

Static cold storage has been the routine method of organ preservation [95]. During the retrieval procedure, the organ is flushed with a perfusion solution and stored at 0 °C to 4 °C on ice. Cooling is intended to suppress metabolic activity [96]. With the increasing use of organs from older donors and organs with reduced organ quality, new storage strategies have been developed for the heart, lung, liver and kidneys [97,98,99,100,101,102].

A recently developed technique of organ preservation is normothermic machine perfusion (NMP), where the graft is perfused with oxygenated blood and nutrients at normothermic body temperature to provide a physiological environment [99]. This emerging technology for organ preservation allows the actual ischemia time to be minimized and the graft function after organ retrieval to be observed and evaluated [103,104]. In addition to the advantages mentioned above, perfusion of the graft offers the possibility of treating and, ideally, improving organ function during storage [105]. There have been efforts to block IRI pathways by inhibitory RNA, to use stem-cell therapy and to treat infections of the graft during NMP [106,107,108]. Another approach to maintaining or even improving organ quality during NMP could be to protect and/or restore the glycocalyx. The following paragraphs present several substances whose glycocalyx-protective properties could be used to treat the graft during NMP.

### 6.1. Albumin

Albumin, the main plasma protein, maintains oncotic pressure, binds to hydrophobic ligands in plasma (e.g., drugs) and is an important buffering system. Importantly, it has been shown that albumin also exerts glycocalyx-protective properties. As a carrier of shingosine-1-phosphate, albumin represents a way to attenuate glycocalyx destruction by shingosin-1-phosphate-mediated inhibition of metalloproteases, which play a central role in glycocalyx degradation [109]. Jacob et al. showed in guinea pig hearts that the glycocalyx is better protected by albumin than by saline or hydroxyethyl starch, measured by the extent of edema formation in the myocardium as an indicator of the vascular barrier function of the glycocalyx [78]. Administration of albumin to the traditional preservation solution “histidine-tryptophane-ketoglutarate” (HTK) resulted in significantly decreased syndecan-1 and heparan sulfate levels in the perfusion solution of transplanted guinea pig hearts after four hours of cold ischemic storage as compared to transplanted hearts perfused with HTK preservation solution [110]. Interestingly, right-heart cardiac output was doubled in hearts perfused with albumin/HTK solution compared to hearts perfused with pure HTK solution. As right-heart failure is a major early complication following heart transplantation, these results underline the importance of the intact glycocalyx in the pathophysiology of heart transplantation [111]. In summary, albumin appears to exert a glycocalyx-protective effect and protect the graft from interstitial edema in animal studies. Since all novel organ perfusion systems require a perfusion solution, the results of this work could be used to consider albumin as a component of an ideal perfusion solution.

### 6.2. Antithrombin

Several studies have shown that antithrombin, one of the most important endogenous inhibitors of coagulation, exhibits glycocalyx-protective properties. Antithrombin interacts with heparin-like glycosaminoglycans, major components of the glycocalyx, thereby promoting the endothelial release of prostacyclin that in turn inhibits leukocyte activation [22]. Besides these anti-inflammatory characteristics, the tight binding of antithrombin to glycosaminoglycans, prevents destructive enzymes from docking to the glycocalyx, which may preserve its function [112]. Several studies have shown that glycocalyx destruction triggered by TNF alpha or by IRI can be prevented by the administration of antithrombin [109,113]. Chappell et al. demonstrated that guinea pig hearts which were pre-treated with antithrombin before a 20 min warm ischemic period, exhibited significantly lower concentrations of glycocalyx degradation products in the perfusate solution than untreated hearts [113]. Taken together, antithrombin appears to be an important factor in glycocalyx protection. In addition to the interaction with the glycocalyx which prevents clotting and inflammation of the endothelium, antithrombin stabilizes the glycocalyx and protects it from shedding.

### 6.3. Glucocorticoids

Glucocorticoids are known to directly inhibit the production of intracellular reactive oxygen species (ROS) [114]. In fact, steroids suppress the expression of metalloproteases, which seem to play a role in the destruction of glycocalyx after trauma and in sepsis [112,115,116]. In an IRI model of an isolated heart, hydrocortisone protected the endothelial glycocalyx, maintained the vascular barrier function and reduced interstitial edema [117]. As glucocorticoids constitute an essential part of immunosuppressive therapy after solid organ transplantation, glycocalyx protection should occur as a positive side-effect. Administration of glucocorticoids during normothermic machine perfusion could result in further protection of the glycocalyx during organ preservation.

### 6.4. Sulodexide

Sulodexide is a glycosaminoglycan composed of approximately 80% heparan sulfate and 20% dermatan sulfate and is approved for the treatment of chronic venous insufficiency and the prevention of recurrent venous thrombosis in certain countries [118,119,120]. In addition to its anticoagulant properties and inhibition of secretion of inflammatory mediators, it mediates the inhibition of heparinase, which in turn cleaves heparan sulfate thereby destroying the endothelial glycocalyx [121,122,123]. It has been shown that in septic mice, where the volume of the glycocalyx is significantly reduced, administration of sulodexide accelerated the recovery of the endothelial glycocalyx, reduced vascular permeability and improved survival rates [23]. In another study, sulodexide reduced the infarct size in mice hearts when administered 30 min after coronary artery occlusion [124]. Although sepsis and IRI present two completely different pathophysiological entities, these results are remarkable as the administration of sulodexide seems to restore the integrity of the glycocalyx, even after the damage has occurred. As damage to the harvested organ occurs during storage and reperfusion, novel therapeutics that improve graft function, even after reperfusion, appear to be a promising approach in transplant medicine to ameliorate acute and chronic graft failure.

### 6.5. Lidocaine

The amid-type local anesthetic lidocaine has been shown to protect the endothelial glycocalyx. A possible explanation for this finding could be the anti-inflammatory properties of lidocaine, which seem to mitigate IRI [125]. Lidocaine mediates its anti-inflammatory properties by blocking ion channels, thus inhibiting the release of cytokines and histamine from mast cells and basophils [126]. In a pig model for left-sided pneumonectomy and auto-reimplantation of the left caudal lobe of the lung, syndecan-1 and heparan sulfate significantly increased in plasma after transplantation [127]. When the animals were pretreated with lidocaine, glycocalyx destruction was attenuated. Intravenous administration of lidocaine also attenuated the rise of matrix metalloprotease 9 and heparinase after reperfusion, indicating reduced glycocalyx-destructive activity. In summary, perioperative administration of lidocaine protects the glycocalyx in animal studies. Whether an already-damaged glycocalyx would benefit from the administration of lidocaine is unclear and needs to be investigated in further studies.

### 6.6. Glycocalyx Components

An intriguing approach to restoring the integrity of the endothelial glycocalyx could be the therapeutic administration of glycocalyx components. In a mouse model of ischemia/reperfusion injury of the cremaster muscle, administration of hyaluronan resulted in glycocalyx repair. Interestingly, this effect was seen regardless of whether hyaluronan was given before or after IRI. This suggests the potential of hyaluronan to prevent glycocalyx destruction, but also its ability to restore the glycocalyx [128]. Other components of the endothelial glycocalyx, e.g., heparan sulfate or sphingosine 1-phosphate, have been successfully used to repair the glycocalyx in cell culture models [129]. The current lack of approval of these substances for human use, however, precludes clinical studies at this time [22].

## 7. Conclusions and Perspective

**Figure 2:**
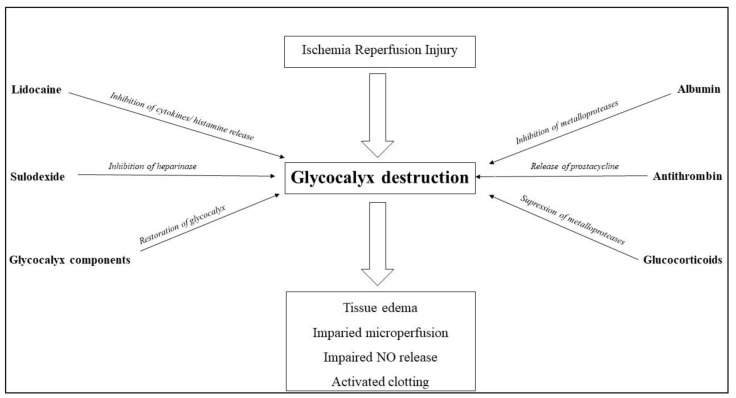
Glycocalyx-protective mechanisms.

Several studies indicate that the integrity and function of the glycocalyx is of prime importance in transplantation medicine. Glycocalyx damage, however, occurs during retrieval, storage and reperfusion of the organ. Even though glycocalyx-damaging mechanisms have been recognized, no therapeutic application has been attempted. Given the circumstances of cold storage, interventions aiming to improve certain organ deficits are technically elusive. Novel organ storage techniques such as normothermic perfusion, however, have been recognized as powerful tools which permit the analysis of organ function during storage. Furthermore, normothermic perfusion enables for drug treatment of organs ex situ. Although various substances with a protective or even regenerative effect on the glycocalyx have been identified, they are not yet used in clinical practice. NMP itself reduces IRI and may, therefore, also be associated with the protection of glycocalyx integrity. Treatment with glycocalyx-protective compounds during NMP may further improve graft quality and function during storage and eventually improve the outcome. Therefore, normothermic machine perfusion not only opens the door to organ function assessment, but may also allow organ therapy. The endothelial glycocalyx is a promising target for such interventions, as its integrity seems essential for physiological organ function. Several pharmacological interventions, such as adaptation of the perfusate to optimize microvascular perfusion of the graft, have already been shown to protect and regenerate the endothelial glycocalyx (Figure 2). Given the importance of glycocalyx protection for transplantation medicine, studies assessing the influence of different compounds on glycocalyx protection and regeneration are warranted.
